# Audit and feedback as a tool to increase compliance with carbapenemase-producing Enterobacteriaceae (CPE) screening and decrease CPE transmission in the hospital

**DOI:** 10.1017/ice.2022.224

**Published:** 2023-11

**Authors:** Orna Ben Natan, Michal Stein, Sharon Reisfeld

**Affiliations:** 1 Infectious Diseases and Infection Control Units, Hillel Yaffe Medical Center, Hadera, Israel; 2 Pediatric Infectious Diseases Unit, Sheba Medical Center, Tel-Hashomer, Israel; 3 Sackler Faculty of Medicine, Tel Aviv University, Tel Aviv, Israel; 4 Rappaport Faculty of Medicine, Technion, Haifa, Israel

## Abstract

**Objective:**

To increase compliance with carbapenemase-producing Enterobacteriaceae (CPE) screening through real-time audit and feedback in our hospital and decrease CPE transmissions.

**Design:**

A before-and-after trial, using active enhanced surveillance of CPE carriers.

**Setting:**

A 500-bed, secondary, university-affiliated hospital that serves a population of 450,000 in a northern district in Israel.

**Methods:**

The study was conducted during 2016–2019 and included patients who were admitted to the hospital and fulfilled CPE screening criteria upon admission and during prolonged hospitalizations. On January 1, 2017, the infection control team implemented a new strategy of real-time feedback toward compliance with in-hospital screening guidelines. Other infection control measurements were performed without interventions. The primary outcome was compliance with appropriate CPE screening. Secondary outcomes included CPE acquisition and compliance with hand hygiene and contact precaution practices. Data were analyzed to calculate differences between compliance with CPE screening during the study period and to test the correlation between contact precautions and hand hygiene practices according to compliance with CPE screening.

**Results:**

During the study period, 3,131 patients were eligible for CPE screening. We detected a statistically significant increase in compliance to CPE screening from 74% during 2017 to 92% in 2018 and 95% in 2019 (*P* < .0001 for both comparisons). We detected a decrease in CPE transmission from 12% in 2017 to 2% in 2019 (*P* < .0001). We did not find any correlation between other infection control interventions and CPE screening and acquisition.

**Conclusion:**

Audit and feedback can improve appropriate CPE screening and may reduce CPE transmission in the hospital.

Infections with carbapenemase-producing Enterobacteriaceae (CPE) have globally increased in recent years and are a major public health concern. Clinical infections have limited treatment options, and infected patients suffer from high mortality rates ranging from 26% to 44%. CPE outbreaks are common, and the control of CPE transmission in hospital settings is a major challenge.^1^ As a “care bundle” to prevent outbreaks, the following practices are recommended: active surveillance and early detection and isolation, placing CPE patients and staff in cohorts, contact precautions, staff education, monitoring compliance to infection control standards, enhanced environmental cleaning and/or decontamination and handwashing interventions.^1^ Screening at admission should include “at-risk” patients, as suggested by Nordmann et al.^2^ The effect of serial screening for CPE during hospital stay, for patients that were not carriers upon admission, is controversial.^3,4^

Audit and feedback comprise an effective tool to improve compliance with clinical practice guidelines, although the effect is variable in different settings and methods used.^5,6^ Real-time feedback improves compliance with hand hygiene,^7,8^ but its role in improving screening practices has not yet been evaluated.

We have described an intervention and its efficacy in decreasing acquisition of CPE in hospitalized patients. Our goal was to increase compliance with hospital guidelines for CPE screening through real-time feedback and, thus, to decrease the rate of CPE transmission in the hospital.

## Methods

### Setting

The Hillel Yaffe Medical Center is a 500-bed, secondary, university-affiliated hospital that serves a population of 450,000 in a northern district in Israel. During the study period, between January 1, 2016, and December 31, 2019, there were ∼45,000 admissions each year.

### Screening and intervention strategy

Patients who were admitted to the hospital and fulfilled CPE screening criteria, as defined by the Israeli National Centre for Infection Control, were screened by rectal swab on admission. All previously known CPE carriers and patients with positive screening results were isolated in a cohort if they were hospitalized in internal medicine wards or in single-bed rooms with contact precautions if they were in other wards.

Starting January 1, 2016, patients were screened weekly if the result on admission was negative and they were still hospitalized, and they were isolated if they became positive. CPE screening was also performed on contacts of newly identified patients. Before the study period, high-risk patients were only screened on admission.

On January 1, 2017, the infection control unit of the hospital implemented a new strategy of real-time feedback on compliance with in-hospital screening guidelines. Since then, 4 times per year, point-prevalence interventions have been performed by the infection control staff. Medical records of all hospitalized patients were reviewed to find those who fit the criteria that justified CPE screening and checked whether they had been properly screened. If patients refused rectal screening, it was documented by the nurse in the specific ward and was analyzed as inappropriate screening. Every quarter, the data were analyzed and sent to the management of the specific ward and to the hospital management as well. In some cases, discussions took place between the infection control unit and the ward.

Other infection control measurements were performed as usual; hand hygiene audits and feedback were performed routinely; and data were analyzed twice per year and sent to the wards and to the hospital management. Compliance with contact precautions and isolation of patients who were known carriers of resistant bacteria (eg, methicillin-resistant *Staphylococcus aureus*, vancomycin-resistant enterococci, Enterobacterales with extended-spectrum β-lactamases) was also monitored weekly by infection control staff, with real-time feedback to the treating personnel, in addition to a biannual report that was sent to the wards and management. All hospital wards were included in the study except for the pediatrics, obstetrics, and the gynecology departments.

Data collection included compliance with CPE screening according to hospital and national guidelines, CPE acquisition and transmission in the hospital, hand hygiene compliance according to infection control observations, and compliance with isolation practices required in different scenarios during hospitalization. Hospital departments were divided into 3 groups: internal medicine, surgical care, and intensive care. Data were analyzed and compared between these 3 groups and the entire hospital, as well as between the years of the study period.

### Laboratory methodology

Surveillance cultures were used to identify and isolate CPE. Swabs were plated onto CHROMAgar KPC media (HyLabs, Rehovot, Israel). Microbiological processing of the cultures for CPE included analysis of every suspicious colony grown on CHROMAgar KPC following 24–48 hours of incubation. Isolates were identified using a Bruker microflex matrix-assisted laser desorption/ionization time-of-flight mass spectroscopy (MALDI-TOF MS) system with the Biotyper 3.1 RTC database (Brukrer Daltonik GmbH, Bremen, Germany). Resistance to meropenem was determined using VITEK2 system (bioMérieux, Marcy l’Etoile, France). Every isolate with meropenem minimum inhibitory concentration (MIC) >0.25 μg/mL was tested for carbapenemase production by multiplex immunochromatographic assay for the qualitative detection of KPC, OXA-48–like, VIM, IMP, and NDM (NG-test CARBA 5, NG Biotech Laboratories, Guipry, France).

### Statistical analysis

The differences in compliance with CPE for different departments during 2016–2019 was calculated using Fisher exact tests. The McNemar test was used for differences within each group of wards. The Spearman ρ correlation was used to test the relation between contact precautions and hand hygiene practices according to compliance with CPE. *P* < .05 was considered statistically significant. SPSS version 25 software (IBM, Armonk, NY) was used for all statistical analysis.

## Results

**Fig. 1.**
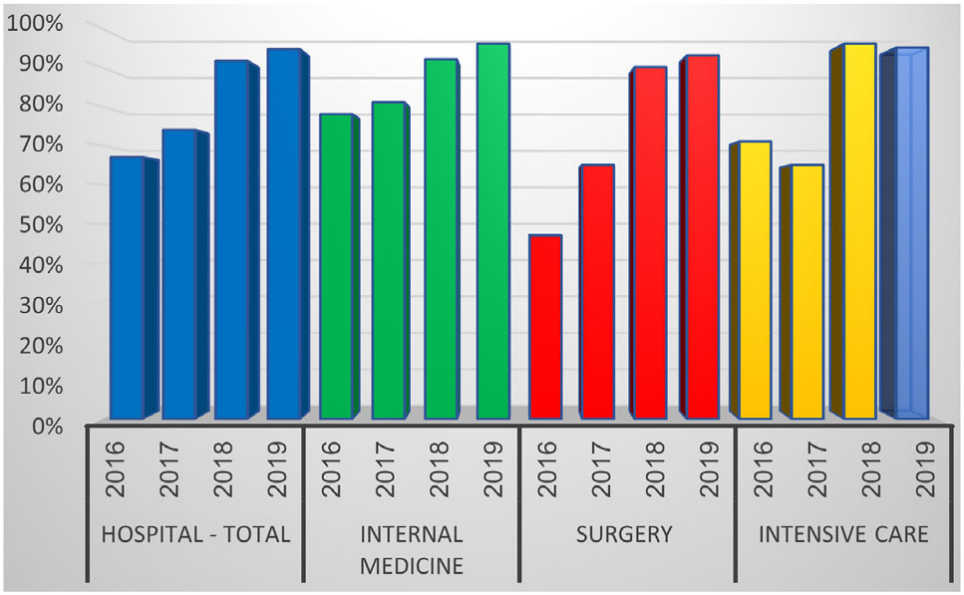
Rate of compliance with carbapenemase-producing Enterobacteriaceae (CPE) screening (percentage from eligible patients according to national and hospital guidelines) during 2016–2019.

During the study period, 3,131 patients were eligible for CPE screening according to hospital guidelines. As shown in Figure 1, there was an increase in compliance with appropriate CPE screening in internal medicine, surgical care, and intensive care wards. For example, during 2016 only 374 patients (78%) in the internal departments were screened for CPE among of 481 who were eligible and should have been screened. In 2019, 470 (96%) of 490 eligible patients were screened. Similar findings in the surgery wards showed that in 2016 only 121 (47%) of 259 patients were screened, but the rate increased to 125 (65%) of 191 patients in 2017 and 309 (90%) of 344 patients in 2018. The difference was statistically significant, especially when comparing 2017 to 2018 and 2019 (Fig. 1).

**Fig. 2.**
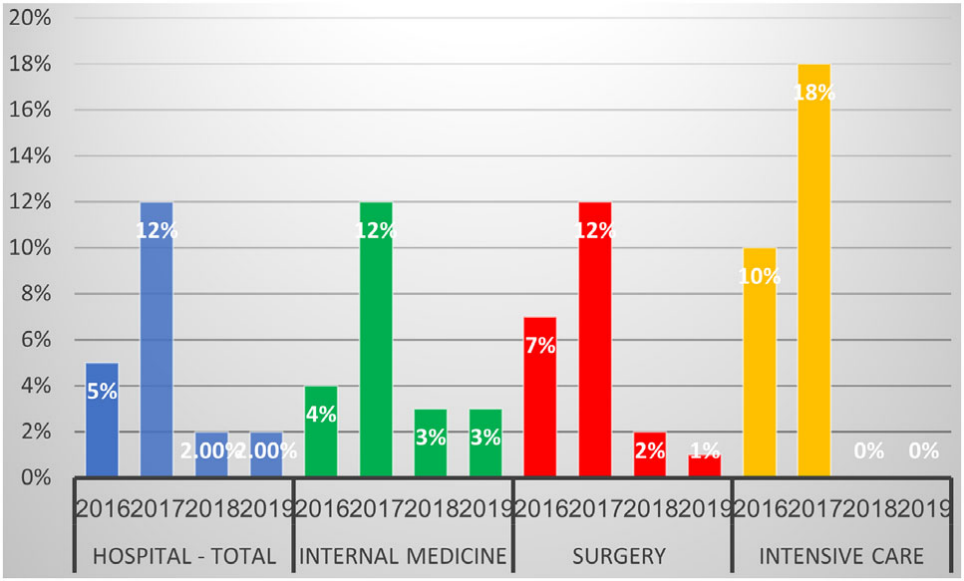
Rate of carbapenemase-producing Enterobacteriaceae (CPE) acquisitions during the study period. Percentage from screened patients, except those who were positive upon admission.

CPE acquisitions during the study period are shown in Figure 2. These are defined as positive CPE screening tests of patients that were repeatedly screened during prolonged hospitalizations or due to contact with another patient. They do not include positive CPE on admission, which were not categorized as in-hospital transmissions. First, there was an increase to 12% CPE acquisitions in the hospital during 2017 compared to 5% during 2016 (*P* = .002), mainly due to a significant increase in the internal medicine departments (*P* = .0006), with no significant increase in the surgery or ICU wards (*P* = .28 and *P* = .0 respectively). Thereafter, a steady decrease to 2% in 2018 (*P* < .0001), the low percentage was sustained in 2019. The results and trends regarding the 3 components (ie, internal medicine, surgical care, and intensive care units) are similar (Fig. 2). During the study period, CPE on admission did not change significantly, except for a slight increase in 2017: 3.3% positivity on admission in 2016, 4.8% in 2017, 2.5% in 2018, and 2.3% in 2019. We also observed some small outbreaks in 2017 in a few wards, but no outbreaks occurred in 2018–2019 (data not shown).

Compliance with hand hygiene practices, as observed and documented by infection control staff, is shown on Table 1. Compliance increased in all 3 groups of wards from 2016 to 2019. As shown in Table 1, compliance across the entire hospital increased from 78% in 2017 to 86% in 2018 (*P* < .001) and remained high in 2019 (85%; *P* = .48). Compliance in the internal medicine departments increased significantly from 72% in 2017 to 84% in 2018 (*P* = .0002) and did not change significantly between 2018 and 2019. Compliance increased significantly in the surgery departments from 80% in 2017 to 87% in 2018 (*P* = .0002) but decreased significantly in 2019 to 82% (*P* = .0021). In the ICUs, we observed an increase in hand hygiene compliance from 83% in 2017 to 88% in 2018 (*P* = .039).

**Table 1.** Rates of Compliance With Hand Hygiene Practices During the Study Period

Variable	2016	2017	*P* Value (2017 vs 2016)	2018	*P* Value (2018 vs 2017)	2019	*P* Value (2019 vs 2018)
**Entire hospital**							
Compliance rate, no. (%)	1,272 (78)	1,071 (78)	.96	3,033 (86)	<.001	2,565 (85)	.48
Sum of opportunities, no.	1,631	1375		3,524		3,002	
**Internal medicine departments**							
Compliance rate, no. (%)	536 (79)	391 (72)	.004	1,470 (84)	.0002	948 (85)	.52
Sum of opportunities, no.	671	537		1,745		1,113	
**Surgical departments**							
Compliance rate, no. (%)	553 (76)	484 (80)	.16	973 (87)	.0002	917 (82)	.0021
Sum of opportunities, no.	719	603		1,115		1,111	
**Intensive care departments**							
Compliance rate, no. (%)	183 (75)	196 (83)	.053	590 (88)	.039	700 (89)	.49
Sum of opportunities, no.	241	235		664		778	

Compliance with contact precautions was consistently high in 2017–2019, with small changes: 94% during 2017 versus 95% during 2018 (*P* < .001) and 92% during 2019 (*P* < .0001) (Table 2). Data were not available for 2016.

**Table 2.** Rates of Compliance With Contact Precautions^a^ During 2017–2019

Variable	2017	2018	*P* Value (2017 vs 2018)	2019	*P* Value (2018 vs 2019)
**Entire hospital**					
Compliance rate with contact precautions, no. (%)	1,586 (94)	882 (95)	<.001	491 (92)	<.0001
Eligible patients for contact precautions, no.	1,687	893		534	
**Internal departments**					
Compliance rate with contact precautions, no. (%)	1,065 (93)	386 (98)	.0003	391 (90)	<.001
Eligible patients for contact precautions, no.	1,142	395		434	
**Surgical departments**					
Compliance rate with contact precautions, no. (%)	453 (96)	446 (99)	.0007	100 (100)	1.00
Eligible patients for contact precautions, no.	470	448		100	
**Intensive care departments**					
Compliance rate with contact precautions, no. (%)	68 (91)	50 (100)	.041	…	…
Eligible patients for contact precautions, no.	75	50		…	…

a No. of patients isolated with contact precautions among eligible patients and percentages.

When testing the relation between contact precautions and hand hygiene practices according to compliance with CPE, hand hygiene compliance trends during the years did not have an effect beyond the effectiveness of CPE screening and feedback on CPE acquisitions during the study period (r = 0.054; *P* = .753). The results were similar in the internal medicine departments and surgical departments: r = 0.403 (*P* = .137) and r = −0.046 (*P* = .870), respectively. In the ICU wards, we detected a marginal correlation between better hand hygiene compliance and fewer CPE acquisitions (r = −0.778; *P* = −.069).

We detected a significant correlation between greater compliance with contact precautions in the hospital and more CPE acquisitions and vice versa (r = .499; *P* = .018). When considering the 3 groups separately, there were no significant correlations for internal medicine departments (r = .354; *P* = .3160), for surgery departments (r = 0.347; *P* = .361), or for intensive care units (r = −0.866; *P* = −.333).

When comparing each year separately and considering hand hygiene compliance and contact precautions and their association with CPE acquisition, there was no significant correlation. The only parameter that was significant was increased contact precautions in 2017, which was correlated with more CPE acquisitions (r = 0.657; *P* = .02).

## Discussion

Based on known methods to increase compliance with hand hygiene and based on real-time audit and feedback, among other methods that were described previously,^7–13^ we developed a new tool for audit and feedback on CPE screening according to national and hospital guidelines. Our aim was to engage hospital personnel with the process to decrease CPE transmissions in the hospital wards.

We started our intervention in 2017, and compliance with CPE screening increased from 67% in 2016 to 74% in 2017 to 92% in 2018 to 95% in 2019, with continued audit and feedback. During the same period, we also observed a decrease in CPE transmission in the hospital wards from 12% CPE transmission in 2017 across the entire hospital to 2% in 2019 (*P* < .0001).

Other infection control measures can also affect CPE transmission; thus, we measured compliance with hand hygiene practices and contact precautions during the study period. Although some changes in hand hygiene compliance were observed during the study period, there was no correlation between them and CPE acquisitions, this is, the relation between hand hygiene and CPE screening and feedback (r = 0.054; *P* = .753). Contact precautions were not related to CPE acquisitions with one exception. Increased compliance was related to more CPE acquisitions in the hospital, which might be explained by finding more CPE carriers via increased screening, which likely reflects the need for greater contact precautions and not the reverse (r = 0.499; *P* = .018).

Previous studies have demonstrated that in many aspects of infectious diseases and infection control, immediate audit and feedback is the most effective intervention to improve work habits. This is true for hand hygiene compliance^7,11^ and for antimicrobial stewardship interventions as well.^14^

Repeat screening for CPE in high-risk patients and prolonged hospitalizations could improve contact precautions and proper isolation when needed and could decrease CPE nosocomial transmission.^3,4^ No one perfect tool can decrease all CPE acquisitions, but a care bundle is similar to other bundles used in infection control interventions.^15,16^ In our study, the association of audit and feedback with decreased CPE transmissions in our hospital was quite remarkable. This intervention may have had an independent effect, regardless of other interventions that were routinely used at the same time, such as contact precautions and hand hygiene compliance.

As far as we know, our study is the first to use real-time audit and feedback regarding appropriate CPE screening, to decrease CPE transmissions in a hospital, and this is the first time a correlation has been demonstrated quite straightforwardly.

This study had several limitations. The study was conducted at a single, medium-sized hospital in Israel; thus, our results might not be generalizable to other hospitals and countries. Nevertheless, the epidemiology and resistance rates observed at our medical center are similar to other medical centers in Israel. Moreover, we used a method that is not specific to certain bacteria or admissions numbers. We did not have data about specific patients, infection control practices, and CPE transmissions by individuals, but this is accepted in similar studies.

The study has some strengths as well. By introducing this method of audit and feedback, we increased the compliance with screening by carriers of CPE and demonstrated its effectiveness. Using Spearman’s ρ correlation analysis, we were able to integrate other possible confounders related to contact precautions and hand hygiene practices and examining their influence on CPE transmission in our hospital.

In conclusion, audit and feedback are important tools to improve appropriate CPE screening to reduce CPE transmission in the hospital. We suggest that this tool be used in as many infection control measures as possible because we know that only a bundle of interventions can make a real difference in infection rates and thus improve patient care.
